# Disrupting the opportunity narrative: navigating transformation in times of uncertainty and crisis

**DOI:** 10.1007/s11625-023-01340-1

**Published:** 2023-06-14

**Authors:** Michele-Lee Moore, Lauren Hermanus, Scott Drimie, Loretta Rose, Mandisa Mbaligontsi, Hillary Musarurwa, Moses Ogutu, Khanyisa Oyowe, Per Olsson

**Affiliations:** 1 Stockholm Resilience Centre, Stockholm University, Stockholm, Sweden; 2 Department of Geography and Centre for Global Studies, University of Victoria, Victoria, Canada; 3 African Centre for Cities, University of Cape Town, Cape Town, South Africa; 4 Southern African Food Lab, Stellenbosch, South Africa; 5 Faculty of Medicine and Health Sciences, Stellenbosch University, Stellenbosch, South Africa; 6 Coracle Consulting, Guelph, Canada; 7 Graduate School of Business, University of Cape Town, Cape Town, South Africa

**Keywords:** Sustainability transformations, Opportunity context, Crisis, Transformative agency, Uncertainty, COVID-19

## Abstract

COVID-19 posed threats for health and well-being directly, but it also revealed and exacerbated social–ecological inequalities, worsening hunger and poverty for millions. For those focused on transforming complex and problematic system dynamics, the question was whether such devastation could create a formative moment in which transformative change could become possible. Our study examines the experiences of change agents in six African countries engaged in efforts to create or support transformative change processes. To better understand the relationship between crisis, agency, and transformation, we explored how they navigated their changed conditions and the responses to COVID-19. We document three impacts: economic impacts, hunger, and gender-based violence and we examine how they (re)shaped the opportunity contexts for change. Finally, we identify four kinds of uncertainties that emerged as a result of policy responses, including uncertainty about the: (1) robustness of preparing a system to sustain a transformative trajectory, (2) sequencing and scaling of changes within and across systems, (3) hesitancy and exhaustion effects, and (4) long-term effects of surveillance, and we describe the associated change agent strategies. We suggest these uncertainties represent new theoretical ground for future transformations research.

**Supplementary Information:**

The online version contains supplementary material available at 10.1007/s11625-023-01340-1.

## Introduction

COVID-19 emerged at a time when both research and advocacy work had already been highlighting the unsustainability, inequities, and injustices in many societal systems (Shi et al. 2021; Kalu and Ott 2019; Tschakert et al. 2019). As the pandemic became a global crisis, the impacts on people’s health and on existing problematic system dynamics was soon apparent. Questions arose about whether the crisis would create opportunities for transformations in these same systems (Berbés-Blázquez et al. 2022; Walker et al. 2020; Pahl-Wostl et al. 2023).

Transformations towards sustainability and justice involve the generation of alternative systems, together with the dismantling or hospicing of problematic systems or sub-system dynamics (Hebinck et al. 2022; de Machado Olivier 2021; Feola et al. 2021; Olsson et al. 2014). Hospicing refers to the care, dignity, and honouring of those who will experience true loss as systems change (de Machado Olivier 2021). The multiple roles that crises play for both the “making and unmaking” (Feola et al. 2021) of transformations have generated much interest (Zakeri et al. 2022; Geels et al. 2022; Benessaiah and Eakin 2021; Schipper et al. 2021; Brundiers and Eakin 2018; Novalia and Malekpour 2020; Loorbach and Huffenreuter 2013; Chapin et al. 2010; Folke et al. 2005), especially since not all crises lead to transformation. Systems can adapt and persist, and sometimes crises can make initial conditions worse (Zakeri et al. 2022; Schipper 2020; Newig et al. 2019; Loorbach and Huffenreuter 2013). Furthermore, a crisis can play out in very different ways across contexts, leading to transformation in one place, and to a “locking in” of unsustainable and unjust systems in another (Geels et al. 2022; Herrfahrdt-Pähle et al. 2020; Novalia and Malekpour 2020; Folke et al. 2005). This indeterminacy makes transformative change work both possible and challenging as crisis unfolds.

Disturbances and uncertainties are understood to be key features of complex systems that cannot be controlled, or entirely predicted (Folke et al. 2016). Thus, change agents interested in transforming such systems will have to grapple with—indeed anticipate—these dynamics at some point. We set out to examine change agents’ experiences of being involved in transformative change efforts as COVID-19 emerged and to explore whether this crisis was an opportunity. More specifically, we explored whether and how a global crisis impacts change agents’ transformative change efforts to move towards sustainability and justice. Our study focused on individuals who were part of a cohort in a programme aimed at applying the latest scientific insights about transformation, complex system dynamics, and social–ecological innovation to their transformative change efforts. We examined how COVID-19 and the various government-led responses impacted their work.

We begin by exploring existing understandings of the relationship between crisis and opportunity for transformations to sustainability and justice, then describe the case study, participants, and methods, and then present our findings. The analysis focuses on the impacts of the responses to COVID-19, four new kinds of uncertainties that emerged, and the change agents’ strategies in navigating these new uncertainties. The pandemic is ongoing and transformations unfold over long periods of time, so we cannot predict how these uncertainties will affect whether transformations occur in the long term. However, we suggest that research needs to better account for these uncertainties in theorizing the processes of transformation, transformative agency, and opportunity—particularly during a crisis.

## The roles of crisis and opportunity narratives in transformations to sustainability and justice

**Fig. 1:**
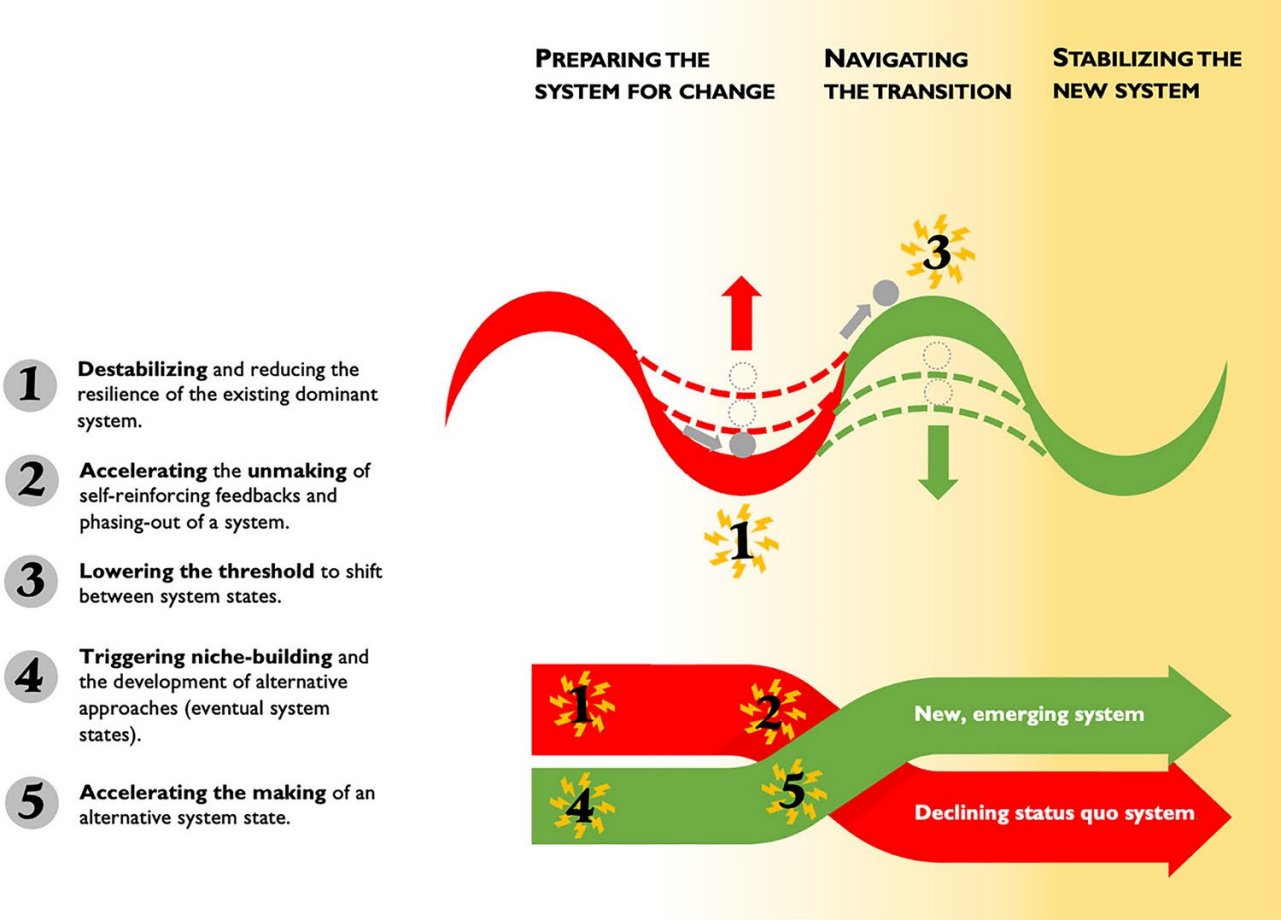
The role of crisis in transformations to sustainability and justice placed on two frameworks from transformations literature: (1) the cup-and-ball model that outlines the phases of transformations processes (Olsson et al. 2006, 2004) and (2) the X-curve, representing the making and unmaking of system dynamics (Hebinck et al. 2022; Loorbach 2014) (illustration by L. Rose)

The roles of crises in transformations are as varied as the crises themselves, whether they are economic, extreme weather events, or involve social–ecological tipping points.^1^ The roles crises can play have been identified across numerous case studies, sometimes using different theoretical frameworks. We synthesize these roles and overlay them on two commonly used frameworks from transformations research (Fig. 1), which are based on defining transformations as fundamentally altering the dynamics or relationships of major structures such as authority, power, and resources, the rules, practices, and processes that reflect and reproduce those structures, the norms, values, and beliefs underpinning the structures and processes, and the way these shape relationships between people, and between people and the planet (Moore and Milkoreit 2020; Olsson et al. 2017). The first framework, the cup-and-ball model (also referred to as multiple basins of attraction) comes from social–ecological systems’ resilience theory and describes transformations as having at least three phases: preparing a system for change, navigating the transition between system states, and consolidating and building the resilience of the new system (Olsson et al. 2006, 2004). The second is the X-curve, which stems from socio-technological system studies and from futures studies which refers to a decline of the dominant system and the surge of a new one that will eventually dominate (Hebinck et al. 2022; Sharpe et al. 2016; Loorbach 2014). Both frameworks illustrate the making and unmaking dynamics that may emerge through crises.

Figure 1 shows how scholarship has identified at least five roles crises can play in transformations (Geels et al. 2022; Hebinck et al. 2022; Herrfahrdt-Pähle et al. 2020; Loorbach 2014; Olsson et al. 2006):

1. destabilizing and reducing the resilience of the existing dominant system,
2. accelerating the unmaking of self-reinforcing feedbacks and phasing out of a system state,
3. lowering the threshold to shift between system states,
4. triggering niche building and the development of alternative approaches (eventual system states), and
5. accelerating the making of an alternative system state.

Crises can destabilize systems and make them more susceptible to change (Cumming and Peterson 2017; Turnheim and Geels 2013). In resilience theory, destabilization involves a reduction in the resilience of the existing system’s state by making the “cup” more shallow and prone to transformation (Fig. 1, point 1). This might include, for example, challenges to a current system’s values, rules, and regulations at multiple levels of society. Scholars have also focused on the strategic agency required to deliberately try to prepare a system for change when such destabilization arises (see phases in Fig. 1) (Westley et al. 2011; Olsson et al. 2004).

Crises can also accelerate the unmaking and phasing out of systems or parts of systems (Fig. 1, point 2). One well-documented example involves the socio-political shocks that may accelerate the unmaking of governance regimes (Herrfahrdt-Pähle et al. 2020). One consequence can be the collapse of either certain aspects or even the entire system (Cumming and Peterson 2017). Examples include the loss of species, the collapse of markets or currencies, or institutional failures (Newig et al. 2019; Boin et al. 2018; Cumming and Peterson 2017). Depending on what aspects of a system are collapsing and for whom, this may be seen as something to be avoided, or something to be facilitated.

Crises may also lower the threshold for transformation to a different system state (Fig. 1, point 3), which may be critical for reaching tipping points (Tàbara et al. 2022; Milkoreit et al. 2018). For example, crisis can lower the resistance to change among key actors and, thus, build support for implementing alternative approaches (Oliver-Smith 1996). Research has documented how the Boxing Day tsunami in 2004 was part of lowering the threshold to transform from a state of conflict to one of peace in Northern Aceh, Indonesia (Brundiers 2020). Threshold lowering may also occur if there is anticipation or fear of a crisis, such as the anticipation of reaching a negative social–ecological tipping point (Herrfahrdt-Pähle et al. 2020; Milkoreit et al. 2018; Biggs et al. 2009; Olsson et al. 2006). Anticipation can motivate a type of proactive, precautionary re-making of systems.

However, crisis narratives that arise in anticipation of events have been widely criticized. The first critique is that such narratives often imply an inevitable, predictable future. This undermines agency and reinforces hopelessness actors may feel in the face of complexity (Evans 2018; Girvan 2017). Second, empirical evidence shows that the reduction of the future to a single state—such as a dystopian future resulting from climate change—requires the oversimplification, understatement, or erasure of multiple contingent processes, such as colonialism, racist imperialism, and exploitative capitalism (Feola et al. 2021; Sharp 2020; Whyte 2018; Girvan 2017). These, in turn, obscure points of intervention and sites of possible change. For instance, Klein (2007) investigated the ways that disasters and crises can be politically manufactured and used to pass exploitive policies. Scholars have argued the importance of understanding the possibilities of crises that *can* emerge, without prescribing their logics of certainty (Evans 2018).

Crisis can also trigger the mobilization of transformative agency to engage in niche-level experiments (Fig. 1, point 4) (Benessaiah and Eakin 2021; Brundiers 2018; Järnberg et al. 2018; Gelcich et al. 2010). For instance, Brundiers (2018) details the strategies that change agents used after the Christchurch, New Zealand earthquake, showing how these helped to see, seize, and sustain opportunities that emerged. Other scholarship has begun to disentangle personal transformations—or the inner journeys—that occur among individuals within these niches, and how these may affect agency and contribute to systemic transformations (Benessaiah and Eakin 2021).

Niche-level experiments can later form the basis of innovations and change at different scales (see Lam et al. 2020; Moore et al. 2015; Westley et al. 2014). Therefore, crisis can contribute to the acceleration of such scaling and rapid transitioning towards new system configurations (Fig. 1, point 5) (Olsson et al. 2004). For example, Herrfahrdt-Pähle et al. (2020) show how alternative governance modes developed in a preparation phase can be accelerated during a navigation phase and later become institutionalized. Transformations may become possible because crises lead to rapid changes in existing structures, processes, practices, values, and narratives, and render the relationships among them more transparent (Fig. 1, point 5) (Benessaiah and Eakin 2021; Schipper et al. 2021; Novalia and Malekpour 2020; Herrfahrdt-Pähle et al. 2020).

The emergence of niche-level experiments or scaling up of previous ones can be considered a relatively positive outcome in moments of crisis. Scholars considering humanitarian aid, disaster relief, and development have also attempted to track whether positive outcomes can be found during crisis (Boin et al. 2018; Agrawal 2011; McSweeney and Coomes 2011; Birkmann et al. 2010). Although the scholarship is nuanced, mainstream discourse has often twisted it to portray the crisis–opportunity relationship with a sense of entrepreneurial optimism. This can tend towards making a business case for finding competitive advantage during crisis by scaling out a specific idea, practice, or product. It may intend to minimize real suffering, but be done without analysing the dynamics that led to the emergence of the crisis (Klein 2007), while tending to assume actors can control aspects of transformational change with the right strategy and good intentions (Leach et al. 2012).

**Fig. 2:**
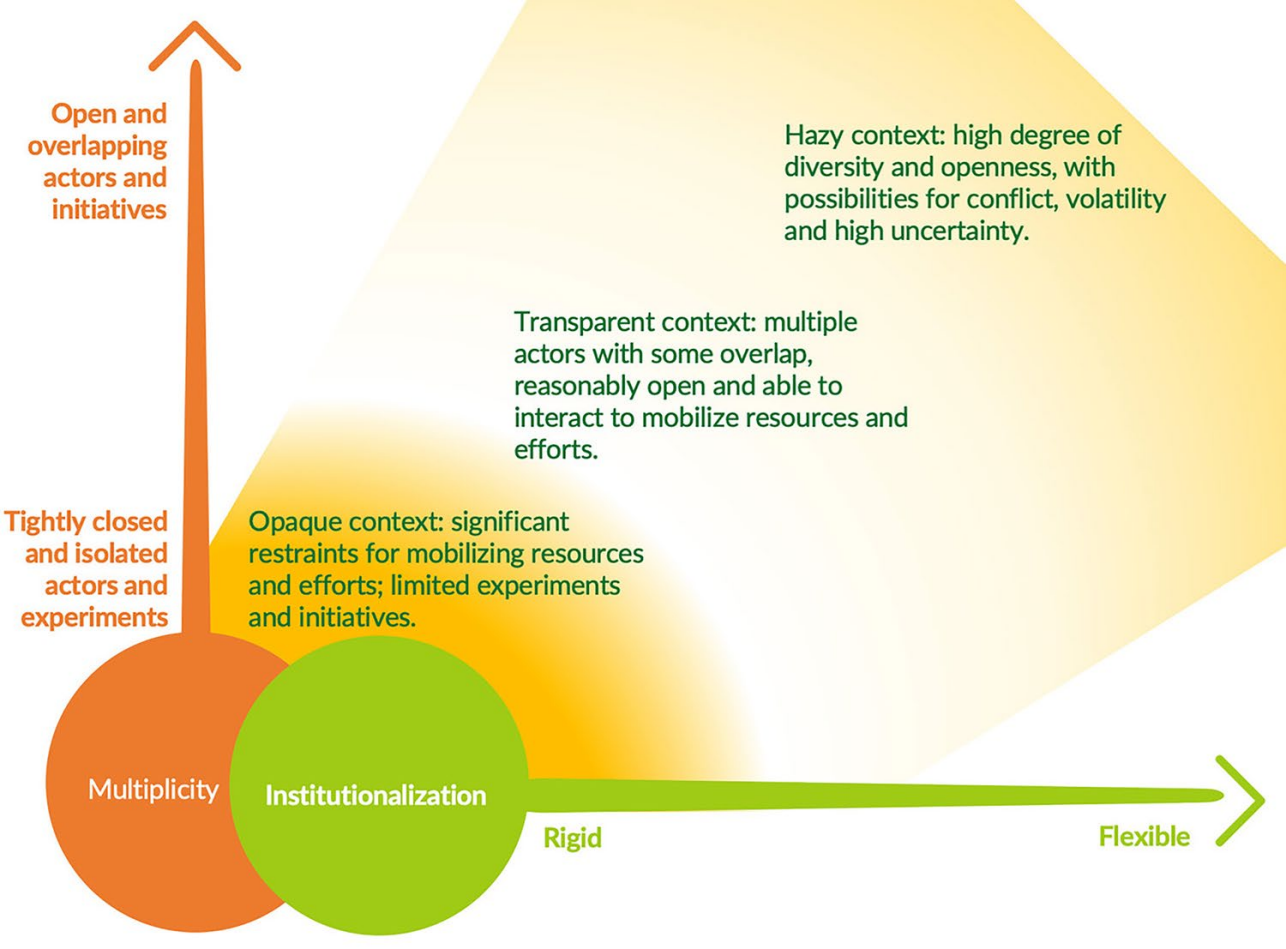
Opportunity contexts can shift between opaque, transparent, and hazy (Dorado 2005). Whether change agents can mobilize resources in these different contexts relates to the multiplicity of actors, organizations, and niche experiments, along with the degree of institutionalization of the field or system (illustration by L. Rose)

Ultimately, each role that crisis may play generates an opportunity context for transformation that may be different from what existed before (Benessaiah and Eakin 2021; Brundiers and Eakin 2018). We use the term opportunity context deliberately, departing from early theorization of transformations that represented crisis as a window of opportunity (see Olsson et al. 2006, 2004; Westley 2002). Rather, we draw on Dorado (2005, p. 387), who studied how change agents continue to work in contexts that may be so uncertain that they do not easily allow for strategic action, but at the same time, do not “comfortably accommodate routine behaviours.” Dorado (2005) contends that opportunity does not stem from actors only finding specific moments to create a political advantage. It also arises from the diversity and plurality of experiments and organizational forms or niches and from the degree of institutionalization and rigidity or adaptiveness in existing regimes (Fig. 2). Such a characterization treats opportunity as always present, not merely a window that is open briefly and then shut.

Dorado (2005) describes the context of opportunity as shifting between opaque, hazy, or transparent across temporal, institutional, and spatial scales (Fig. 2). Opaque contexts exist where entire fields of work are either isolated or highly institutionalized to the point of rigidity, and the mobilization of new combinations of resources or different resources altogether becomes nearly impossible (Dorado 2005). Hazy contexts have a multiplicity of institutions, and circumstances are considered volatile and uncertain, whereas transparent contexts have multiple opportunities for actors to mobilize agency and resources to create new arrangements (organizational or otherwise) and gain support for social innovations (Dorado 2005). Westley et al. (2013) build on this in their analysis of how transformative agency can shift and respond to these opportunity contexts. They illustrate how novelty and innovation can emerge in any social–ecological context, albeit under differing constraints. Other scholarship has demonstrated how system features and capacities related to social memory, cultures of risk taking versus aversion, and learning can all play key roles in defining the opportunity context (Herrfahrdt-Pähle et al. 2020; Novalia and Malekpour 2020; Birkmann et al. 2010).

In summary, crisis can play several roles in the making and unmaking dynamics of transformation, and questions of crisis for whom and by whom, and transformations for whom and by whom, are relevant across these different roles. As we were interested in the experiences of change agents who were attempting to transform system dynamics, we explored whether and how COVID-19 responses impacted their efforts for systemic change and whether there were patterns in how the pandemic played out across different contexts required investigation.

## Methods

Empirical evidence about the potential role of crisis in transformation has generated much interest, but existing studies have mostly focused on national crises or disaster events, exploring whether new transformations resulted. How a larger-scale crisis might affect existing efforts to transform systems remains open and, therefore, this study was exploratory in nature. Consequently, the study was conducted using a grounded theory approach following Strauss and Corbin (1990) and focused on change agent efforts in six countries in Africa.

### Case study: Transforming Change Program

We focused on individuals who comprised a cohort of the Transforming Change Program, which was designed and delivered through a partnership with the Stockholm Resilience Centre, Southern Africa Food Lab, and How We Adapt South Africa funded by Swedish Institute. Participants were recruited through an open nomination process, with nominators recognizing the nominees’ work to challenge existing problems and to transform systems in efforts to achieve the sustainable development goals in the African context. Once nominated, if they were interested in learning and applying the science of transformations, complex systems, and resilience, they could apply. The cohort included 18 participants from six countries: Ghana, Kenya, Nigeria, Rwanda, South Africa, and Uganda, and their work cut across different domains (see Table 1).

**Table 1:** Participant areas of work and focal areas for transformation

Country	Area of work	Transformative change efforts focused on:
Ghana	Environmental management	Flood reduction
	Environmental management	Wetlands management and ecological services
	Gender	Climate vulnerabilities and economic opportunities
	Sanitation and waste management	Resources and knowledge mobilization for healthy futures
	Transportation	Fair and sustainable urban mobility solutions
Kenya	Energy	Renewable energy access and women’s economic empowerment
	Energy	Clean energy solutions and gender equity
	Livelihoods	Rural livelihoods and inclusion
	Livestock management systems	Climate change, pastoralist livelihoods, gender equity
Nigeria	Agriculture	Cross-generational transmission of sustainable agricultural practices
	Education	Early childhood education in a context of wholistic family and community support
	Employment and inclusion	Fair, meaningful work for the marginalized and disadvantaged
	Employment and Inclusion	Advocacy, livelihoods, and policy change for people living with disabilities
South Africa	Education	Fair and accessible higher education
	Employment	Youth employment and sustainable economic opportunities
	Leadership	Leadership in development and during transitions
	Urban development	Violence prevention
Uganda	Employment	Workforce development with youth
	Energy	Renewable energy and agriculture nexus

The programme involved one online module and two in-person modules over five months. The final in-person module was cancelled due to COVID-19 and was re-organized online. Participants wanted to spend sessions hearing from each other about how they were handling the restrictions in their work. Therefore, the facilitation team offered to conduct this study and interview people to understand if there were patterns in approaches and impacts. This meant participants were not selected through a randomized process. The researchers had established relationships with participants, and the entire group (delivery team and participants) had co-produced knowledge and terminology for discussing participants’ work, transformation, complex systems, and resilience.

### Data collection and analysis

Data collection involved semi-structured interviews during June–August 2020, with 15 of the 18 participants, and a survey of 177 media articles about COVID-19 in the participants’ six countries. The media review included media articles, blogs, and cross-references with government press releases, registered national media houses, and grey literature reports (such as WHO or UN reports) published between September 2019 and November 2020. The data collection included dates prior to the pandemic because of related events that occurred earlier than 2020, but were exacerbated by COVID-19—for example, locust invasions in Kenya and Uganda. Three team members conducted the interviews, while an additional four supported media collection and data coding. With such a large team, we used several approaches to ensure data robustness and coding accuracy. Team members collaboratively developed the interview guideline, whether they would conduct interviews or not. Those conducting interviews met after the first set and discussed the guideline and emerging themes to ensure we were testing theories as they emerged. The other four transcribed the recordings, two coded the interviews, and two coded the media articles. Codes were detailed in shared documents that all team members could contribute to and were discussed at regular meetings. Emerging themes were tested throughout data collection, and media search terms were adjusted as codes emerged (e.g. initial terms included the country name and COVID-19, and later included country name, COVID-19, and hunger or food security). The final selective coding process of all data was done by the lead author. A critical aspect of grounded theory is testing until theoretical saturation is achieved. While no new themes were emerging by the end of the interviews and media analysis, we acknowledge we were limited in how many interviews we could hold due to the number of participants in the programme. This may affect the robustness of our theory, and we suggest further research to test and build upon the findings.

## Results

We explored whether and how this global crisis impacted change agents' transformative change efforts to move towards sustainability and justice. Participants in this study were initially confronted with only the anticipation, rather than the wide spread of the virus in their countries, since sizeable outbreaks in Africa lagged far behind Europe, and North and South America. Yet, all countries where participants were based had official national responses to the crisis. One participant noted that this is not typically the case for issues that they might characterize as crises, such as droughts or locust invasions.

Our findings revealed at least three changes to the conditions and contexts of participants’ work—all of which interacted. These changes and consequences included: economic, hunger, and gender-based impacts. Due to the high levels of uncertainty about the virus itself, the top-down and highly institutionalized responses, the increased constraints and competition for resources, and the inability to easily convene and socially organize during lockdowns, we suggest that COVID-19 represented a different kind of crisis than has been studied previously in transformations scholarship. We then analyse four new uncertainties that emerged: (1) robustness of preparing a system to sustain a transformative trajectory, (2) sequencing and scaling of changes within and across systems, (3) hesitancy and exhaustion effects, and (4) long-term effects of surveillance. We also observed and analysed the change agents response strategies to these uncertainties, which included: repurposing and bridging actors and organizations to cope and adapt to the uncertainties, adjusting “conceptual closure” and re-thinking and re-scaling approaches, and navigating the psychological aspects of the uncertainties within their systems. We suggest these findings may represent new theoretical ground to consider in future sustainability transformations research.

### Lockdown

In response to COVID-19, lockdown or shelter-in-place orders were established by governments worldwide. While quarantines, social distancing, and other measures have a long history as tools for managing outbreaks and diseases (Rothstein 2015), the large-scale use since 2020 is unprecedented (Buheji et al. 2020). Restrictions included geographic containment (travel restrictions, closing borders), closures and prohibitions (e.g. restrictions on public gatherings, school closures, closure of non-essential shops or services), and home confinements (curfews, self-isolation for those infected or at risk of exposure, and stay-at-home orders) (see Haider et al. 2020). In Ghana, Kenya, Nigeria, Rwanda, and South Africa, several of these restrictions were put in place within days or weeks of the first cases being diagnosed (Adebowale et al. 2021; Afriyie et al. 2020; Amzat et al. 2020; Haider et al. 2020; Kenya Ministry of Health 2020). In Uganda, restrictions on public gatherings were in place four days before the first case was reported in the media (see Birner et al. 2021; Uganda Ministry of Health 2020) (see Supplementary Table 1).

Restrictions tended to ease and then come into effect again in different ways in different countries (Birner et al. 2021; Haider et al. 2020; Musanabagnwa et al. 2020) and continued to do so after the data collection. Three key impacts emerged from these restrictions, which changed the conditions for participants’ work: economic impacts, hunger, and gender-based violence and other gendered impacts. Participants were now navigating and needing to respond to these evolving conditions—sometimes instead of, or in addition to, their previous efforts.

### Changing conditions and consequences: economic impacts

The economic implications of COVID-19 were mentioned by every participant. They appeared to be related to at least three issues: (1) unemployment levels, (2) the precarity of individual incomes and the ways COVID-19 amplified how far they needed to be stretched, and (3) the longer-term uncertainty regarding funding for participants’ organizations and the communities they served.

At national levels, unemployment soared, with an estimated three million becoming unemployed in South Africa in only a short time (e.g. Nwosu and Oyenubi 2021) and 1.7 million lost jobs March–August 2020 in Kenya (KNBS 2020). In May 2020, unemployment in Rwanda reached its highest point since 2016 (NISR 2021), and the Nigerian National Bureau of Statistics estimated 20% of the full-time workforce in Nigeria lost their jobs in 2020 (UNDP & NBS 2021). In Ghana, 42,000 were laid off March–August, with wages reduced for an additional 770,000 workers (approximately 25.7% of the workforce) (World Bank, Statistical Services Ghana, UNDP 2020).

None of the participants interviewed were unemployed at the time of the study but the national unemployment levels affected their work. For instance, one participant’s work focused on youth unemployment in the context of high structural unemployment rates. Their organization set up work experiences in partnership with the private sector and government. The youth they served were suddenly competing with millions of others unemployed, all of whom had work experience that the youth did not. Organizations they had partnered with in the past were now sometimes closing, or unable to create new openings. The organization tried to create more limited types of labour that still contributed to their mission, such as having youth temporarily employed in sewing COVID face masks.

Another participant’s organization had always focused on students in marginalized situations, or on those who faced challenges in completing their studies. Some students were sending scholarship money back to their families prior to COVID-19. Now, with soaring unemployment, these students were supporting even more:

“…I know several students that we support more than once a month. I know that there's a family of eight in both situations and that student’s bursary is the only income for that family.” (Participant 7)

Many government agencies re-allocated resources to COVID-19 responses for health care or for immediate needs such as food relief (see Supplementary Table 1). These re-allocations increased uncertainty about long-term funding:

“…with the governments that we work with, everyone is so focused on the now. And so there's this massive scramble to cut budgets left, right, and centre to make so much more budget available for food relief. And for COVID 19 relief. And no one's thinking about what is the long-term strategy…” (Participant 2)

However, many government efforts were not necessarily perceived as helpful by participants. Some mentioned that money did not always reach the people it was intended for, because the government did not have an effective distribution system (Nathan and Benon 2020). Sometimes, re-allocations created restrictions on partnerships, engagements, and existing funds, and participants anticipated this would have enduring negative consequences on the communities they served. The strategies to navigate this uncertainty varied, with two participants describing a more aggressive pursuit of grants than they might typically seek, and another deliberately engaging donors in decisions about how to respond to unfolding conditions in communities they supported.

Unusually, one participant experienced investors being more open than before to providing financial support. This participant worked in renewable energy and found that interest by government and banks in stimulating the economy for recovery meant that they were seeking opportunities and were interested in sustainable energy investments. In the longer term, this could contribute to a transition for energy supply in rural regions, but the resources and authority remained with dominant actors. There was little to indicate that the full social–ecological consequences around energy transitions were being considered. Moreover, starting new projects was nearly impossible because travel restrictions did not allow participants to go to communities.

### Changing conditions and consequences: hunger

Hunger is a multi-dimensional issue, linked to many aspects of the social, ecological, and economic structures that existed prior to COVID-19, as well as to government pandemic responses (Nwosu and Oyenubi 2021; Ssali 2020). A direct link can be made to increased unemployment and the loss of purchasing power. This affected both families that could no longer purchase adequate food, and those who relied on selling it. Hunger was exacerbated by closing borders and restrictions of movement which disrupted local, regional, and global food supply chains (Berbés-Blázquez et al. 2022). Participants highlighted that in Kenya, pastoralists could no longer sell and trade livestock at local markets, while in Nigeria, the closing of food markets disproportionately affected women who were the predominant sellers (see also Ssali 2020). One participant noted that increases in prices combined with panic buying may have contributed to food shortages.

Hunger meant people were not able to engage with training, education, or other initiatives intended to strengthen capacity for change efforts. As one participant described:

“There was a huge drop in the training, not because of the distancing…but it was more to do with: there's no money, there's no food. And I can't come to the training unless I'm getting some money to go and feed my children.” (Participant 15)

Participants also pointed to the fact that government-led responses to hunger, which often came in the form of emergency food kits, missed the most vulnerable and missed addressing the systemic drivers of hunger—which participants had already been trying to change (Nathan and Benon 2020):

“There's a big food crisis in South Africa at the moment because of COVID. Even when we shift our focus to a very specific thing like hunger, we're still not focusing on the systemic issue of hunger; the fact that we create a society where someone needs to have a job in order to feed themselves, in order to access the basic constitutional right to food…And…we're not going to address these things, because those things require time, they require relationships, they require thoughtful consideration, and no one is in that space right now.” (Participant 2)

“One thing that’s clear is that COVID-19 has exposed the inability (of government) to have data about people with disabilities, as well as people who are vulnerable and exposed…it then shows to us government never knows who are the people that are vulnerable. And they don’t even know where they are. So, palliatives (food packets) were distributed. But not on the basis of needs! But on the basis of location… ‘Oh, we’ve been able to distribute to 10,000 people, 12,000 people and a 100,000 people,’ but not on the basis of those who need it…” (Participant 9)

In Kenya and Uganda, participants were concerned about the simultaneous locust invasion and flooding, as this combination of events would threaten already food insecure people (see also Birner et al. 2021). One organization was trying to create a social-political early warning system that would identify hotspots for potential conflict. The hope was this would become a means to institutionalize a form of preparedness related to hunger and more. They continued to engage government to ensure that drought, locusts, and COVID-19 impacts gained attention at both local and national levels.

### Changing conditions and consequences: gender-based violence

Throughout the period of this study, increased rates of gender-based violence were reported. Numerous reasons can relate to and intersect with the dynamics of increased violence (Barasa et al. 2021; Uzobo and Ayinmoro 2021), which help explain the relationship to a pandemic. Exact numbers are difficult to ascertain given that violence is often unreported or not legally recognized, and participants did not cite specific numbers. However, rates were reported to have increased by: 500% in South Africa March–June 2020 (Roy et al. 2021, see also Metsing 2020), and 149% in Nigeria March–April 2020 (Roy et al. 2021; see also UN Women 2021). Reports indicate that in Kenya, calls to a national hotline increased in the first two months of lockdown by 775%, and an additional 3650 cases were reported between March and July 2020. Reporting to Ugandan police on gender-based violence tripled in April 2020 compared to April 2019 (Roy et al. 2021). Additionally, lockdown measures and rhetoric of pandemic and disease were associated with increasing vulnerability of LGBTQIA2S + communities (Al-Ali 2020).

Several participants were focused, at least in part, on trying to change systems related to women’s rights, employment, participation in decision-making, and leadership in governance, and in supporting the education of women or girls.^2^ They expressed concerns that there would be a long-term decrease in girls’ enrolments following government ordered school closures, reversing the trend that had been in place.

“…this COVID situation, which has made a lot of children not be in school, has also implications on the number of girls getting pregnant. And if the government decides to say all children should be back in school now, I’m very sure we’re going to have a serious reduction in the enrolment of girls from elementary to secondary.” (Participant 3)

The expected decrease was linked to concerns that pregnancies would increase because of rape, transactional sex due to unemployment and hunger, or simply due to more time spent with partners (see also Ssali 2020). Participants said they expected marriages of younger girls to increase.

Perhaps, most concerning was the nonlinear nature of responding to gender inequalities. Several stated that even if government reopened schools, businesses, and clinics, they did not expect enrolment, pregnancy, or gender-based violence rates to return to pre-pandemic levels.

## New uncertainties for transforming systems during crisis

Some conditions described above existed before COVID-19 and, in fact, were issues many were already trying to transform. However, the responses had an amplifying effect, rapidly increasing unemployment, hunger, and gender-based violence. These increases, and the need to mobilize coping and adaptive capacities for the changing circumstances, raised at least four new kinds of uncertainties for transformations to sustainability and justice: uncertainties around (1) robustness of preparing a system to sustain a transformative trajectory, (2) sequencing and scaling of changes within and across systems, (3) hesitancy and exhaustion effects, and (4) long-term effects of surveillance, and required change agents to alter their strategies.

1. Robustness of preparing a system to sustain a transformative trajectory

Preparing to transform a system and preparing to respond to a crisis are two different activities. However, evidence from this study indicates that the former may help the latter in some instances because of the capacity change agents have created for strategies of: redundancy, repurposing, and bridging between otherwise disconnected groups. For example, one organization had invested significant effort over several years to build local planning and development committees that were not initially recognized as formal governance structures. These were not created to focus on health or pandemic related issues. However, during COVID-19, they became important sources of institutional capacity that could share information about transmission, government responses, and recommended health measures such as social distancing and masks. This points to the capacity created by relationships of trust that could be mobilized and re-purposed. Re-purposed community organizations could ensure COVID-19 responses were at least partly shaped by locals, who might not otherwise have a voice in the top-down crisis processes.

Although some organizations lost staff and revenue—especially those engaged in transportation, employment, or tourism—those that were able to mobilize with a different purpose could at least maintain their operations (see Table 2).

**Table 2:** Examples of strategies used by participants’ organizations to cope and adapt to the impacts of COVID-19

Shifting from in-person training to virtual policy advocacy.
Shifting from focusing only on drinking water supply and sanitation to include irrigation, to address food insecurity.
Changing group sizes for convening sessions; convening more frequently in smaller groups.
Shifting toward employment opportunities for youth in COVID response initiatives.
Shifting from visiting communities to sharing information by radio dispatch.
Changing safety protocols.
Lobbying to be named as an essential service to keep operating.

Despite these adaptations and response strategies by change agents, there were uncertainties about previous work, which was perceived or hoped to be transformative at a local scale, and whether the impacts would be robust throughout COVID-19. Significant concerns were raised about the long-term implications of intersecting gender (in)equality and economic (in)security. After several years of increasing enrolment in schools in certain communities or training women to be entrepreneurs and sell their own goods to gain livelihood security, these trends were expected to experience major regressions—even after COVID-19 measures were lifted. As one participant stated: “*What a lot of work we have done towards increasing enrolment, towards ensuring that girls, you know, move higher! And all of this [pandemic] has come to bring us back.*”

Other participants were concerned about losing momentum with project and partnerships, while restricted from visiting communities or other countries:

“… we were building this momentum, there's a project going on, people are having hope this will happen. But, when this crisis comes, then people start to move on with a new normal and finding ways to cope. And my fear is that we will have to start from zero in terms of motivating people or encouraging them to participate.” (Participant 15)

2. Sequencing and scaling within and across systems

Participants noted that the vulnerabilities revealed by COVID-19 across multiple systems illustrated that the work they had been doing, while important, may not have been as transformative as they expected. They began to re-think their approaches, which implied an additional function of crisis for change agents’ strategies: to interrupt what we refer to as “conceptual closure” on the part of systems actors, reminding change agents of the long-term and nonlinear nature of transformative work, which has no static end point. For instance, two participants engaged in renewable energy access in rural communities reflected that getting every household connected was a less urgent priority than ensuring that the hospital has an energy supply for oxygen machines. Similarly, the previous focus for some on increasing school enrolments or creating opportunities for un- and underemployed people were questioned now that schools were closed and job losses increased. These initiatives presupposed the achievement of levels of energy access, education access, and employment.

Others described a form of re-scaling their efforts. One participant had been working mainly in surrounding countries and was suddenly full-time in their home community. Lockdown changed where they focussed their efforts and which challenges they sought to address. Others recognized they had been scaling out their initiatives—that is, repeating it in different contexts, hoping this added up to a larger, systemic transformation. They realized they were approaching the change process without recognizing the capacities for change and innovation within the communities in which they worked, and without considering how to scale up. One noted that their entire way of operating needed to change, even when restrictions of movement were lifted, if their work was to contribute to a transformation. Another recognized that efforts to scale were completely constrained because of the lockdown measures and restrictions. This participant was working to transform the transportation system through a centralized rideshare platform. The aim was to better address the needs of rural and peri-urban populations who travel long distances for work and health care, etc., while simultaneously reducing traffic, congestion, and pollution. Their work halted entirely during the time of this study, but they noted single ridership with international service companies increased—the opposite of what was hoped for. Scaling their work was blocked because of the way their competitor, an international company already large in size, could exploit the new conditions. There was a re-thinking of what would contribute to community transformation and how, and of the possibilities that now existed or had been constrained.

3. Hesitancy and exhaustion effects

As noted, one participant reported that COVID-19 created new investor interest and opportunities in sustainable energy development. Another believed that the significant changes in the ways people were working might create opportunities because people would recognize their capacity to make big changes in a short period of time.

All of the remaining participants were more hesitant to ascribe to COVID-19 any sort of opportunity for transformations that would be sustainable and just. They highlighted that, while COVID-19 might have revealed or reinforced that many systems or dynamics needed to change, there was little appetite among government or funders for the risk of taking on something new. The rationale was that new initiatives or approaches would also inevitably entail other new problems and issues, for which there was little capacity.

Participant interviews showed a long list of psychological effects: anxiety, depression, ennui, confusion, loneliness, a sense of abandonment, and a “mood killer.” While these were individual experiences, it raised questions about how COVID-19 might affect capacities for transformation longer term. However, participants reflected on their own coping strategies to accept that the context had changed, with several participants describing how they were juggling child care, and education of children, while moving their organizations’ work entirely online. One participant explained, *“…part of it is just how to deal with adverse circumstances, knowing how to keep going on in the process, not letting it get to you as much as it might…You can see an opportunity and not be able to take advantage of it. That doesn't mean that you didn't see the opportunity or that you suck…”.*

Overall, all participants continued with concrete actions and our findings did not indicate a major erosion of the participants’ capacities to at least try to continue their work.

4. Long-term effects of surveillance

To ensure lockdown and stay-in-place orders, many countries pursued a variety of enforcement tactics. While militaries have often played a role in technical or logistical support during humanitarian crises or disasters, the enforcement of COVID-19 restrictions set new precedents for sustained militarized responses in non-war contexts—most often in cities—to control the movement of people (see Gibson-Fall 2021). Reports indicate murder, abuse, rape, and punitive control of civilians during lockdowns in all six countries, as well as others around the world (Namwaya 2020; Obaji Jr. 2020; Okech et al. 2020; Olewe 2020; Parker et al. 2020; Rampedi et al. 2020). Uncertainty existed about whether this would legitimize increased police brutality and militarization for the long term.

One participant noted: “*Twelve black people died during our first 30 days of lockdown at the hands of our police and our Defence Force. Twelve people!*” Yet, narratives of violence and safety placed emphasis on civilians’ and households’ behaviours rather than scrutinizing how countries’ own policing systems were committing violence. Reports also indicated increased exposure to violence of already systemically vulnerable and marginalized groups. As medical anthropologists Levine and Manderson (2020) stated, *“As protests spread worldwide, examples of police brutality of Black South Africans began to appear on video and social media posts, and two forms of breath restriction and death converged and overlapped: Black people around the world not only feared and were most likely to die from the virus; they also feared and are most likely to be killed by security officers.”*

Some surveillance was related to data mining and online monitoring that was part of contact tracing and quarantine enforcement, but it was unclear if this information was being used beyond just managing the virus. However, while these longer-term concerns were in the media and documented in scholarship (Birner et al. 2021; Maza 2020; Okech et al. 2020; Transnational Institute 2020), participants did not mention or say how this might affect their own work.

## Discussion

We discuss the role of COVID-19 as a crisis and whether it contributed to the making and unmaking processes of transformations to sustainability and justice. We also discuss the opportunity contexts that emerged and examine the implications of the uncertainties that arose, and which remain today.

### COVID-19 as a crisis

This study revealed that some aspects of COVID-19 were distinct from other crises covered in previous transformations research, including: (1) the global scale, (2) the concurrent national responses, and (3) social distancing which hindered the ability to physically meet and convene, combined with the length and repeated instances of restrictions across multiple waves.

The global nature of the crisis mattered in participants’ work because the major economic and hunger impacts, along with the new uncertainties they created, were the result of imposed restrictions to prevent spread from other countries, disrupted global supply chains, and cross-scale economic linkages. At the time of data collection, they were not the consequence of the virus spreading in the continent. In this way, fear or anticipation drove narratives that did not create opportunity for transformation but instead led to top-down, command and control responses that reinforced existing dominant systems.

While our data was collected in 2020, the experiences of the author team and ongoing media reports indicate that these impacts have remained. For example, while economic recovery packages offered by government in South Africa are no longer called COVID relief, they continue because of the ongoing economic challenges. Although not observed during data collection, economic reprioritization in high-income countries is expected to change the funding landscape for the kind of work participants are undertaking.

As one participant noted, the national level of response to the crisis was unusual, since other shocks and crises had not received the same attention. National responses happening in multiple countries simultaneously created conditions not typically observed when a single country responds to a crisis. Nearly every system on every continent experienced disruption. Crises can play a role in destabilizing systems; but in this case, the *responses* to the crisis may have destabilized some dominant systems, such as food systems. Both the crisis and responses also destabilized the alternative systems that participants were trying to build; that is, they destabilized the making processes of transformation.

The inability to convene, physically travel to communities where participant’ worked, or meet with social networks in person, created constraints. Previous research has pointed to the importance of social networks during crisis for collaboration, informal support, learning, and knowledge building (Benessaiah and Eakin 2021; Novalia and Malekpour 2020; Boin et al. 2018; Birkmann et al. 2010). Several participants described how COVID-19 restrictions created limitations, also shaped by who had access to internet, who could afford data, who could access applications and software, and who had time outside the caregiving duties created by pandemic conditions. This limited capacities that could have been mobilized. Our study showed that participants continued to adapt and learn, but it was in spite of these conditions, not because there were benefits to them. We did not observe new types of niche-building, although attempts to maintain, re-purpose, and bridge previous niches did occur.

We note that the repeated waves of restrictions following our data collection period, the disinformation campaigns and limited access to vaccines, and other global events (e.g. severe drought, heatwaves, and Russia’s invasion of Ukraine) contributed to worsening food and energy poverty (Zakeri et al. 2022). Thus, it is difficult to attribute specific impacts to the pandemic alone, since these issues became entangled both in time and space, showing compounding, as well as cascading, crises.

### Opportunity context: from hazy to opaque

We did not observe clear positive progress that improved sustainability and justice; rather, there were ongoing indications of worsening inequality—as other studies have begun to confirm (Geels et al. 2022; Merkle et al. 2022; Zakeri et al. 2022; Fagbemi 2021). Based on the Dorado (2005) framework, one might expect that any crisis would contribute to a hazy opportunity context since, by definition, such a context is volatile and uncertain. Our data showed numerous instances where the opportunity context could be described as opaque with highly institutionalized responses and enforced constraints that reduced multiplicity.

It could be argued that some participants attempted to generate an opportunity context that was transparent by making it obvious to those around them and to governments how non-COVID issues were connected to the pandemic and needed to change. We hypothesize that in these instances, there may be possibilities if the context shifts to hazy or transparent, ensuring that capacity for the broader change work is maintained. However, many participants believed their efforts went unnoticed or struggled to get the attention of those with power and resources—showing how an opaque context can be an isolating experience.

### Emerging uncertainties

The four uncertainties that emerged raise new insights and questions for transformations scholarship.

#### Robustness of preparing a system to sustain a transformative trajectory

The making aspects of a transformation process were reversed in some instances, highlighting the precarity of outcomes that can often seem transformative in the short term. This may be especially true if these processes are too closely intertwined with aspects of the dominant system—especially precarious dominant systems—that will need to go through unmaking. As an example, evidence currently shows that both the global financial and food systems are not expected to be robust or sustainable in the face of numerous global social–ecological changes (Crona et al. 2021; Willett et al. 2019). Therefore, transforming practices or structures toward securing someone’s livelihood or securing rights for women to participate in what are known to be highly volatile and vulnerable systems, inevitably comes with massive uncertainty and risks. Our findings suggest that scholars and practitioners interested in transformations may need clearer criteria to identify whether and under which conditions an initiative has transformative potential or whether, in the long term, it will perpetuate vulnerabilities, injustices, and unsustainability of the dominant system. Finally, it reinforces the need to evaluate transformative change in short, medium, and long-term time frames.

#### Sequencing and scaling of changes within and across systems

Participants reflected on whether they had tried to resolve complex social–ecological challenges by over-emphasizing a single solution or approach and neglecting other steps that may have been needed. We hypothesize that practitioners reflecting on these questions may be more responsive to changing dynamics. Although further exploration will be needed, we suggest a sixth role for how crisis may affect transformations, based on how COVID-19 changed how participants and their organizations understood the complex system dynamics shaping their problems in ways that might not have been possible before. We refer to this role as a disruption of “conceptual closure” because of the way it opens up understandings of what or how transformation is occurring. This disruption may lead change agents to make decisions that alter the future trajectory and strategies of the transformation they are seeking to enable. Alternatively, they may recognize that their initiatives are necessary but insufficient. They may not have enough agency to shift the practices, structures, and resource flows necessary to meaningfully reorient their work. These insights might then require research and advocacy that specifically targets other actors such as development agencies and funders. All this emphasizes the importance of the distributed nature of agency and systems entreprenership (as opposed to focusing on single change agents or entrepreneurs) and how that distribution is shaped by different opportunity contexts (Westley et al. 2013).

#### Hesitancy and exhaustion effects

Previous research has documented the strategies and cognitive, structural, and agency features that can help change agents to see, seize, and sustain opportunities following crises (Benessaiah and Eakin 2021; Herrfahrdt-Pähle et al. 2020; Brundiers 2018). Evidence in our study highlighted how, even if opportunities existed, the combination of restrictions and the enormous toll on mental health and well-being eroded capacity in the system in ways that resulted in a non-transparent opportunity context. Brundiers (2018) also noted the exhaustion and defeat that change agents experienced after a honeymoon period of intense activity and hope, due to the long-term nature of transformative change. Perhaps worryingly, our data did not indicate any honeymoon had occurred before these sentiments became real for participants. We suggest that efforts will be required to support healing, rejuvenation, inspiration, connection, and sensemaking for change agents in both the short- and long-term, knowing that these are critical capacities for both enduring crisis (Oliver-Smith 1996) and for any transformation process (de Machado Olivier 2021; van der Merwe et al. 2020; Moore et al. 2018).

#### Long-term effects of surveillance

Questions arise about the impacts of increased (and increased legitimacy for) surveillance and brutal enforcement. This raises concerns and questions for people who are trying to change the status quo of dominant, unsustainable, inequitable, and unjust systems. Evidence shows that change agents seeking to shine a light on unsustainable practices and injustices around the world are at great risk (e.g. Global Witness 2021; Scheidal et al. 2020). Combining surveillance with top-down, command and control responses may make some decisions clearer in the moment; but this does not necessarily equate to a transparent opportunity context. Longer-term, increased surveillance has the potential to make opportunity contexts more opaque and much more risky. Lines are blurry between disease monitoring and individual surveillance, with many risks regarding how data collected on people’s movements and social contacts could be used (see, for example, French and Monahan 2020).

## Conclusion

We set out to explore whether and how change agents continue to transform systems toward more sustainable and just configurations by disrupting problematic dynamics or generating possible alternatives during times of crisis. Although previous studies have highlighted at least five roles that crises could play in transformations, our study confirms that crisis does not guarantee a transparent opportunity context. Furthermore, we suggest there is a sixth role of crisis in relation to change agents and their agency that has not been previously identified. A crisis can interrupt conceptual closure in actors seeking to change systems, affecting how change agents consider temporal dynamics, evaluate the durability of change, and plan sustained efforts. This could lead to new and reimagined planning and actions or to a greater understanding of the limitations of agency.

COVID-19 lockdowns and restrictions had an amplifying effect on specific system dynamics, increasing the magnitude and severity of poverty, hunger, and gender-based violence and inequality. Pandemic responses also created new uncertainties that could change how we understand the role of crises and the opportunity context for transformative agency. These uncertainties included the robustness of previous change in sustaining a transformative trajectory; the sequencing and scaling of changes; confusion and hesitancy effects that make it difficult to determine which circumstances represent opportunities; and possible new risks posed by increased surveillance, police brutality, and militarized responses. Although the focus of our study is on the six countries in which the participants were based, these trends are expected to be relevant elsewhere.

In conclusion, the “crisis as opportunity” narrative is significantly challenged by the experiences and events examined in this study. The oversimplification of the complex dynamics of systems does injustice to the opacity, uncertainty, and challenges precipitated by crisis. At the same time, change agents do identify, shape, and respond to opportunities within these hazy and opaque contexts, applying different strategies with an eye on both short-term adaptations and longer-term transformations. These strategies included repurposing and bridging actors and organizations to cope and adapt to the uncertainties, and re-thinking and re-scaling approaches, and navigating the psychological aspects of the uncertainties within their systems. Our research suggests that the result of change agents’ efforts ensured that the capacities, trust, and nascent institutions that had been developed as part of efforts to transform systems prior to COVID-19, could be mobilized to ensure that responses to this crisis might at least be done differently than in the past. We close by urging that these ongoing efforts not be ignored, and that the longer-term risks posed by the new and emerging uncertainties continue to be examined.

## Supplementary Information

Below is the link to the electronic supplementary material.

Supplementary file1 (PDF 92 KB)

## Data Availability

All publicly available data on government responses has been cited in text and in the supplementary information. As per standard practices in research ethics, interview data cannot be shared openly to protect study participant privacy.

## Footnotes

1 Some scholars treat crisis and disaster as distinct, with crisis representing a threat, and disaster involving the threat playing out as an episodic event that causes measurable damage and suffering (Boin et al. 2018). Others recognize this distinction as problematic, given the longer-term social, political, and economic underpinnings of any disaster (Novalia and Malekpour 2020). Consequently, it has become common to use them interchangeably in transformations scholarship. Here, we use the term crisis to recognize the protracted nature of COVID-19, which is not a single episodic event, and the cascading nature of the pandemic (Galaz et al. 2011).

2 We recognize gender plurality, and that broader efforts are being made to reflect both plurality and intersectionality within gender-based programming within development. However, we want to ensure we speak to the concrete lived experiences of participants and in these cases, the projects primarily focused on cis-women and girls, and therefore we use these categories to reflect the contexts that were being examined.
