# Supplementary material for: Disrupting the opportunity narrative: navigating transformation in times of uncertainty and crisis
Source: Sustain Sci. 2023 Jun 14:1–17. Online ahead of print. doi: 10.1007/s11625-023-01340-1 (PMC10265562; doi:10.1007/s11625-023-01340-1)
Supplement: Supplementary file 1 — Supplementary file1 (PDF 92 KB) [file 11625_2023_1340_MOESM1_ESM.pdf]

Supplementary Table 1. Examples of lockdown restrictions and policy responses during COVID-19 in six countries: Ghana, Kenya, Nigeria, Rwanda, South Africa, and Uganda

Country	Date first Covid-19 case reported	Examples of Restrictions	Sources
**Ghana**	March 12, 2020	**Measures to control transmission**<br>• The government initially imposed a full ban on all public gatherings, including conferences, workshops, funerals, festivals, political rallies, church activities and other related event. Some activities were later allowed but with controls such as limits on number of participants.<br>• Closure of schools, and higher learning institutions, and beaches.<br>• Restrictions on travelers from to Ghana from regions with a higher number of COVID-19, except for citizens and residents.<br>• Closure of all land and sea borders and suspension of passport services<br>• Partial lockdown of Accra and Kumasi cities except for essential service providers.<br>• Mandatory use of masks was to be enforced by businesses and organization.<br>• Expanding healthcare facilities, e.g., construction of additional Intensive Care Unit (ICU) bed facilities in key regions/cities.<br>**Socioeconomic policy responses**<br>• Government provided three months of free water to citizens, from April to June 2020, to encourage hygiene and handwashing.<br>• Ghana Parliament established a national Trust Fund to receive and manage Covid-19-related funds, including donations from individuals, corporate bodies.<br>• Tax waivers on income taxes of GHS174 million cedis (equivalent to US$30 million) for front line health workers.<br>• Financial support to eligible small business under the CAP - Coronavirus Alleviation Program Business Support Scheme.<br>• Provision of relief finance through the ministry of Education to support private schools who were affected by COVID-19 induced shutdown of schools.<br>• Provision of food aid to vulnerable populations.<br>• Provision of subsidised electricity during the initial days of the pandemic	(Afriyie et al., 2020; Amewu et al., 2020; Arhinful and Opoku, 2020; Haider et al., 2020; Human Rights Watch, 2020; Kenu et al., 2020; World Bank, Statistical Services Ghana, UNDP, 2020)

**Kenya**	March 12, 2020	**Measures to control transmission:**<br>• These included, timed curfews (e.g., dusk to dawn), travel ban (both local and international), closures of schools, places of worship, and workplaces, restrictions on social gatherings such as weddings and funerals, and encouraging remote work for both private and public agencies where possible.<br>• Mandating increased health service capacity, supplies and expanded mass testing.<br>• Release of prisoners serving sentences for petty offences - about 4,800 prisoners were released in April 2022.<br>• Restrictions on number of passengers on public transport systems<br>**Socioeconomic policy responses:**<br>• Extension of civil servant health insurance to all county-level health workers; provision of cash and food aid to vulnerable communities/families.<br>• Reduction of tax burden on citizens through the Tax Law (Amendment) Act, 2020. The act reduced personal and resident corporate income tax rate from 30% to 25%, provided complete tax relief to low-income earners, reduced the turnover tax rate for small and medium-sized enterprises from 3% to 1%; and lowered the value added tax (VAT) rate from 16% to 14%.<br>• The National Treasury provided additional Ksh10B (equivalent to US$100M) to support the elderly, orphans, and other vulnerable groups with cash transfers.<br>• Implementation of policies to encourage cashless transactions such as mobile money and credit cards. For example, The Central Bank issued a directive reducing the commission charged on mobile money transactions.<br>• Part of IMF's Rapid Credit Facility	(Barasa et al., 2021; Birner et al., 2021; Gentilini et al., 2020; Jerving, 2020; Kansiime et al., 2021; Kenya Ministry of Health, 2020; Kiruga, 2020; KNBS, 2020; McDade et al., 2020)
**Nigeria**	February 27, 2020	**Measures to control transmission**<br>• Training volunteers to share accurate information about COVID-19 in their communities.<br>• Suspension of international travel; restrictions on inter-state travel across the country (e.g., movement from Abuja, Delta, and Lagos states); closure of schools, and higher learning institutions, countrywide restriction/closure of social gatherings, including religious activities, bars, restaurants and cafes, non-essential shops, recreational parks and facilities, home confinement and or dusk-to-dawn curfews.<br>**Socioeconomic policy responses**<br>• Implementing the Emergency Economic Stimulus Bill 2020 to provide support to businesses and individual businesses such as	(Adebowale et al., 2021; Amzat et al., 2020; CDC Nigeria, 2020; Dixit et al., 2020; George, 2020; President Buhari, 2020; UNDP & NBS, 2021)

		50% tax rebates to businesses to allow them to keep paying workers employed at the time.<br>• Cash transfers of up to 20,000 Naira ($52USD) to poor and vulnerable households registered with the National Social Register (NSR).<br>• Stimulus package by the Central Bank of Nigeria which provides a credit of 3 million Naira to families impacted by COVID-19.<br>• Food assistance to vulnerable families in some states such as Lagos, and Ogun states	
**Rwanda**	March 14, 2020	**Measures to control transmission:**<br>• Curfews (e.g. 8PM to 5.AM) , restrictions on local (rural to urban and vice versa) and international travel, mass screening and testing, encouraging electronic payments and online banking services where possible, closure of markets or restrictions on number of vendors, work from home policy for private and public employees, restrictions on social gatherings such as weddings and funerals (for example, gatherings not exceeding 30 persons).<br>• Hand washing, mask wearing and social distancing.<br>• Closure of schools, places of worship, and closure or restriction on operating times for bars, hotels, restaurants, and recreational centers such as gyms.<br>• Restrictions on number of passengers on public transport systems<br>**Socioeconomic policy responses:**<br>• Senior government officials forfeited their April 2020 salary, contributing the funds to state efforts to COVID-19.<br>• Provision of food relief to vulnerable households in Kigali and other urban centers; instituting zero charges on mobile money transfers and lifting limitations on the number of daily transactions possible through mobile money, and implementation of other social protection measures.<br>• Easing loan repayment conditions to borrowers, and other measures to ease financial pressure on citizens and businesses by commercial banks.<br>• Introducing a fund to support access to capital for small enterprises.<br>• The Rwanda Revenue Authority (RRA) extended the deadline for businesses to file and pay income taxes and relaxed other administrative requirements.	(African Development Bank, 2020; Bower et al., 2020; IMF, 2020a; Ministry of Finance and Economic Planning, 2020; Musanabagnwa et al., 2020; NISR, 2021)

**South Africa**	March 5, 2020	**Measures to control transmission**<br>• Declaration of a national state of disaster; restrictions on local/domestic travel and social gatherings; closure of schools, colleges and universities, non-essential shops, restaurants, bars and cafes, recreational parks, and facilities; restriction on sale of alcohol and tobacco products; closure of workplaces and encouraging remote work; imposition of regional or nationwide curfews; and ban on international travel on international flights, closure of land and sea borders<br>**Socioeconomic policy responses**<br>• Issuance of a special unemployment insurance benefit under the Unemployment Insurance Fund for contributors to the fund who have lost income due to the pandemic<br>• Social assistance programmes such as cash transfers and food aid<br>• Provision of tax relief including a delay of income tax remittances by small businesses<br>• Debt relief fund to provide relief on existing debts and repayments for small businesses<br>• Introduction of an employment tax incentive aimed at reducing youth unemployment by encouraging employers to hire young job-seekers	(de Villiers et al., 2020; Goitom, 2020; Heiberg and Winning, 2020; IMF, 2020c; KPMG South Africa, 2020; Nwosu and Oyenubi, 2021; Public Service and Administration South Africa, 2020; South African Government, 2020)
**Uganda**	March 21, 2022	**Measures to control transmission**<br>• Total ban on transportation, in both private and public vehicles<br>• Closures and Restrictions on business operations except for essential businesses, closure of schools, closure of airports except for Cargo, closure of bars, restaurants, cafes, recreational parks and facilities and religious and other social and cultural facilities.<br>• Nationwide curfew – later restricted travel/movement times<br>**Socioeconomic policy responses**<br>• Increased health spending and strengthened social protection for most vulnerable<br>• Bank of Uganda lowered interest rates<br>• Created a Rapid Credit Facility with IMF<br>• Instituted cost recovery for COVID tests for certain groups<br>• Food assistance to vulnerable people in Kampala (actual numbers remain difficult to confirm)	(Athumani, 2020; Birner et al., 2021; IMF, 2020b; Ladu, 2020; Mahmud and Riley, 2021; Margini et al., 2020; Nathan and Benon, 2020; UNOCHA, 2020)

References for Supplementary Table 1

Adebowale, A.S., Fagbamigbe, A.F., Akinyemi, J.O., Obisesan, O.K., Awosanya, E.J., Afolabi, R.F., Alarape, S.A., Obabiyi, S.O., 2021. The spread of COVID-19 outbreak in the first 120 days: a comparison between Nigeria and seven other countries. BMC Public Health 21, 1–8.

African Development Bank, 2020. Rwanda - Covid-19 Crisis Response Budget Support Program (RCRBS) [WWW Document]. URL https://projectsportal.afdb.org/dataportal/VProject/show/P-RW-K00-013 (accessed 12.19.22).

Afriyie, D.K., Asare, G.A., Amponsah, S.K., Godman, B., 2020. COVID-19 pandemic in resource-poor countries: challenges, experiences and opportunities in Ghana. The Journal of Infection in Developing Countries 14, 838–843.

Amewu, S., Asante, S., Pauw, K., Thurlow, J., 2020. The Economic Costs of COVID 19 in Sub Saharan Africa: Insights from a Simulation Exercise for Ghana (No. STRATEGY SUPPORT PROGRAM , WORKING PAPER 52). IFPRI.

Amzat, J., Aminu, K., Kolo, V.I., Akinyele, A.A., Ogundairo, J.A., Danjibo, M.C., 2020. Coronavirus outbreak in Nigeria: Burden and socio-medical response during the first 100 days. International Journal of Infectious Diseases 98, 218–224.

Arhinful, E., Opoku, D.M., 2020. Parliament approves GHS174M tax waiver for frontline health workers. Citinewsroom - Comprehensive News in Ghana.

Athumani, H., 2020. Uganda Cuts Cost of COVID Test From $65 to $50. VOA News.

Barasa, E., Kazungu, J., Orangi, S., Kabia, E., Ogero, M., Kasera, K., 2021. Indirect health effects of the COVID-19 pandemic in Kenya: a mixed methods assessment. BMC Health Services Research 21, 1–16.

Birner, R., Blaschke, N., Bosch, C., Daum, T., Graf, S., Güttler, D., Heni, J., Kariuki, J., Katusiime, R., Seidel, A., Senon, Z.N., 2021. 'We would rather die from Covid-19 than from hunger'-Exploring lockdown stringencies in five African countries. Global Food Security 31, 100571.

Bower, J., Apell, D., Twum, A., Umulisa, A., 2020. Rwanda's response to COVID-19 and future challenges. IGC. URL https://www.theigc.org/blog/rwandas-response-to-covid-19-and-future-challenges/ (accessed 12.19.22).

CDC Nigeria, 2020. CDC Supports Nigeria to Stop the Spread of COVID-19 I CDC [WWW Document]. URL https://www.cdc.gov/globalhealth/stories/2021/nigerias-effort-to-stop-covid.html (accessed 12.19.22).

de Villiers, C., Cerbone, D., Van Zijl, W., 2020. The South African government's response to COVID-19. Journal of Public Budgeting, Accounting & Financial Management 32, 797–811. https://doi.org/10.1108/JPBAFM-07-2020-0120

Dixit, S., Kofoworola Ogundeji, Y., Onwujekwe, O., 2020. How well has Nigeria responded to COVID-19? Brookings Institute. URL https://www.brookings.edu/blog/future-development/2020/07/02/how-well-has-nigeria-responded-to-covid-19/ (accessed 12.19.22).

Gentilini, U., Almenfi, M., Orton, I., 2020. Social Protection and Jobs Responses to COVID-19: A Real-Time Review of Country Measures. World Bank Report.

George, L., 2020. Nigeria to extend coronavirus lockdowns for 14 more days: President Buhari. Reuters.

Goitom, H., 2020. South Africa Government Measures to Contain the Spread of COVID-19 and Mitigate Damages | [WWW Document]. In Custodia Legis: Law Librarians of Congress. URL //blogs.loc.gov/law/2020/04/south-africa-government-measures-to-contain-the-spread-of-covid-19-and-mitigate-damages/ (accessed 12.19.22).

Haider, N., Osman, A.Y., Gadzekpo, A., Akipede, G.O., Asogun, D., Ansumana, R., Lesselis, R.J., Khan, P., Hamid, M.M.A., Yeboah-Manu, D., Mboera, L., 2020. Lockdown measures in response to COVID-19 in nine sub-Saharan African countries. BMJ Global Health 5, e003319.

Heiberg, T., Winning, A., 2020. South Africa to impose 21-day lockdown as coronavirus cases jump. Reuters.

Human Rights Watch, 2020. Ghana: 1st Covid-19 Case in Psychiatric Hospital. Human Rights Watch. URL https://www.hrw.org/news/2020/04/30/ghana-1st-covid-19-case-psychiatric-hospital (accessed 12.19.22).
IMF, 2020a. Rwanda Harnesses Technology to Fight COVID-19, Drive Recovery. International Monetary Fund News.
IMF, 2020b. IMF Executive Board Approves a US$491.5 Million Disbursement to Uganda to Address the COVID-19 Pandemic. International Monetary Fund News.
IMF, 2020c. Policy Responses to COVID19 [WWW Document]. IMF Policy Responses to COVID 19. URL https://www.imf.org/en/Topics/imf-and-covid19/Policy-Responses-to-COVID-19 (accessed 12.19.22).
Jerving, S., 2020. Cash transfers lead the social assistance response to COVID-19 [WWW Document]. Devex. URL https://www.devex.com/news/sponsored/cash-transfers-lead-the-social-assistance-response-to-covid-19-96949 (accessed 12.19.22).
Kansiime, M.K., Tambo, J.A., Mugambi, I., Bundi, M., Kara, A., Owuor, C., 2021. COVID-19 implications on household income and food security in Kenya and Uganda: Findings from a rapid assessment. World Dev 137, 105199. https://doi.org/10.1016/j.worlddev.2020.105199
Kenu, E., Frimpong, J.A., Koram, K.A., 2020. Responding to the COVID-19 pandemic in Ghana. Ghana Med J 54, 72–73. https://doi.org/10.4314/gmj.v54i2.1
Kenya Ministry of Health, 2020. First case of coronavirus disease confirmed in Kenya (Statement). Kenya.
Kiruga, M., 2020. President Kenyatta takes pay cut, imposes curfew to halt coronavirus. The Africa Report.com.
KNBS, (Kenya National Bureau of Statistics), 2020. Quarterly Labour Force Report, Quarter 2 (No. Quarter 2).
KPMG South Africa, 2020. South Africa - KPMG Global [WWW Document]. KPMG. URL https://home.kpmg/xx/en/home/insights/2020/04/south-africa-government-and-institution-measures-in-response-to-covid.html (accessed 12.19.22).
Ladu, I.M., 2020. Double tragedy facing economy: Covid-19 and Elections. Ugandan Daily Monitor.
Mahmud, M., Riley, E., 2021. Household response to an extreme shock: Evidence on the immediate impact of the Covid-19 lockdown on economic outcomes and well-being in rural Uganda. World Development 140, 105318. https://doi.org/10.1016/j.worlddev.2020.105318
Margini, F., Pattnaik, A., Jordanwood, T., Nakyanzi, A., Byakika, S., 2020. Uganda's Emergency Response to the COVID-19 Pandemic: A Case Study. ThinkWell and Ministry of Health Uganda., Washington DC, USA and Uganda.
McDade, K.K., Ogira, D., Onyango, J., Ojal, J., Kokwaro, G., Mao, W., Yamey, G., 2020. Kenya's Policy Response to COVID-19 – The Center for Policy Impact in Global Health. The Centre for Policy Impact in Global Health, North Carolina, USA.
Ministry of Finance and Economic Planning, 2020. Businesses affected by the Impact of COVID-19 to receive EUR 55 million financing boost [WWW Document]. URL https://www.minecofin.gov.rw/news-detail/businesses-affected-by-the-impact-of-covid-19-to-receive-eur-55-million-financing-boost (accessed 12.19.22).
Musanabagnwa, C., Munir, L., Mazarati, J.B., Muvunyi, C.M., Nsanzimana, S., Mutesa, L., 2020. Easing lockdown restrictions during COVID-19 outbreak in Rwanda. Rwanda Public Health Bulletin 2, 24–29.
Nathan, I., Benon, M., 2020. COVID-19 relief food distribution: impact and lessons for Uganda. Pan Afr Med J 35, 142. https://doi.org/10.11604/pamj.supp.2020.35.142.24214
NISR, (National Institute of Statistics of Rwanda), 2021. Labour Force Survey Trends, November 2020, Q4 (No. Quarter 4). National Institute of Statistics of Rwanda.

Nwosu, C.O., Oyenubi, A., 2021. Income-related health inequalities associated with the coronavirus pandemic in South Africa: A decomposition analysis. International Journal for Equity in Health 20, 1–12.
President Buhari, 2020. COVID-19: Nigeria extends lockdown by two weeks in Lagos, Abuja & Ogun States [WWW Document]. CNBC Africa. URL https://www.cnbcafrica.com/2020/covid-19-nigeria-extends-lockdown-by-two-weeks-in-lagos-abuja-ogun-states/ (accessed 12.19.22).
Public Service and Administration South Africa, 2020. State of Disaster: Guidelines for the Containment/Management of the Coronoa Virus (COVID 19) in the Public Service.
South African Government, 2020. Regulations and Guidelines - Coronavirus COVID-19 | South African Government [WWW Document]. URL https://www.gov.za/covid-19/resources/regulations-and-guidelines-coronavirus-covid-19 (accessed 12.19.22).
UNDP & NBS, 2021. The impact of COVID-19 on business enterprises in Nigeria. United Nations Development Program and Nigerian National Bureau of Statistics.
UNOCHA, 2020. Uganda Key Message Update: Floods and COVID-19 restrictions lead to an increase in the population in Crisis (IPC Phase 3), May 2020 - Uganda | ReliefWeb (Famine Early Warning System Network updates). United Nations Office for the Coordination of Humanitarian Affairs.
World Bank, Statistical Services Ghana, UNDP, 2020. How COVID-19 is affecting firms in Ghana: Results from the Business Tracker Survey.
